# Toughened High-Flow Polypropylene with Polyolefin-Based Elastomers

**DOI:** 10.3390/polym11121976

**Published:** 2019-12-01

**Authors:** Xiong Wang, Sheng Hu, Yi Guo, Guangquan Li, Renwei Xu

**Affiliations:** 1 Lanzhou Petrochemical Research Center, Petrochemical Research Institute, PetroChina, Lanzhou 730060, China; guiyi2@petrochina.com.cn (Y.G.); liguangquan@petrochina.com.cn (G.L.); xurenwei@petrochina.com.cn (R.X.); 2 College of Chemical and Chemical Engineering, Xi′an Shiyou University, Xi’an 710065, China; husheng0927@126.com

**Keywords:** polypropylene, polyolefin-based elastomer, polyolefin elastomer (POE), olefin block copolymer (OBC), propylene-based elastomer, toughening effect

## Abstract

Polyolefin is the most widely used and versatile commodity polymer. In this work, three types of polyolefin-based elastomers (PBEs) were adopted to toughen a high-flow polypropylene to improve its overall performance. The chain microstructures of these PBEs, including ethylene/1-octene (E/O) random copolymer from Dow Chemical′s polyolefin elastomer (POE), olefin block copolymers (OBCs) of E/O from Dow, and ethylene/propylene random copolymer from ExxonMobil’s propylene-based elastomer, were elucidated by GPC, ^13^C NMR, TREF, and DSC techniques. The mechanical, thermal and optical properties, and morphology analysis of the PP/PBE blends were also studied to investigate the toughening mechanism of these PBEs. The results showed that all three types of PBEs can effectively improve the Izod impact strength of the PP/PBE blends by the addition of the rubber compositions, at the cost of the stiffness. PBE-1 and PBE-2 were found to have a great stiffness–toughness balance with about 1700 MPa of flexural modulus, about 110 °C of HDT and 3.6 kJ/m^2^ of impact strength on the prepared PP/PBE blends by forming separated rubber phase and refined spherulite crystals. As a result, the OBC with alternating hard and soft segments could achieve a similar toughening effect as the E/P random copolymer. Surprisingly, no obvious rubber phase separation was observed in the PP/PBE-4 blend, which might be due to the good compatibility of the E/P random chains with the isotactic PP; therefore, the PP/PBE blend obtains great toughness performance and optical transparency with the highest Izod impact strength of 4.2 kJ/m^2^ and excellent transparency.

## 1. Introduction

Polyolefins are the most widely used and versatile commodity polymers, and their properties vary from plastic to elastomer [1,2,3,4]. Since the discovery of Ziegler-Natta catalysts for olefin polymerization in the 1950s, the production of polyolefins with various chain microstructures and properties has continuously grown with rapid development of catalyst technology combined with polymerization process innovation [5,6,7,8,9,10,11,12,13,14].

Polypropylene is undoubtedly one of the most robust material fields in the polyolefin production and consumption market globally [15,16,17,18], with a current annual demand of about 56 million tons in 2016 [19]. They are used in a wide range of applications ranging from packaging to lightweight engineering plastics for automobile, electrical and electronics, construction, medical, equipment, and facilities industries [20,21]. China now possesses the largest market share in PP production of above 22 million tons. Due to rapid market expansion of takeout for dining box and automobile industries in China, the high-flow homo polypropylene market has also witnessed a dramatic increase to about above 600 kilotons annually in the recent few years.

Although the high-flow homo polypropylene with high melting index (typically above 50 g/10 min) and good processability possesses high flexural modulus, the impact strength is relatively low compared to the ethylene/propylene copolymer and easily suffers from brittle fracture [22]. In order to solve this problem, two approaches are typically adopted to improve the overall performance of the polymer. One alternative is to introduce an extra operation line for the incorporation of a small amount of ethylene into the isotactic chain in the PP production facility, and the other method is to make post-modifications of the high-flow polypropylene by blending elastomer with PP, and glass fibers for automobiles [23,24,25]. 

The Polyolefin-based elastomers (PBEs) have received considerable attention because of their low density, recycling potential, better chemical resistance, processing advantages, and good resilience without permanent deformation. Unlike rubber, they do not require vulcanization. In addition, the low cost together with the wide availability of ethylene, propylene and α-olefin monomers makes the polyolefin-based elastomers more desirable. The ethylene/1-octene random copolymers (POEs) are a typical class of PBEs, and they are produced by Dow Chemical′s constrained geometry catalyst (CGC) metallocene catalysts in a solution process. Due to the single-site nature of CGC, they have a much narrower short chain branching (SCB) distribution than the Ziegler-Natta catalysts. In contrast, Exxon has developed the propylene-based ethylene/propylene elastomers using metallocene catalysts by the Exxpol™ technology.

Recently, ethylene/1-octene multiblock copolymer (OBC) has been commercialized successfully using Dow Chemical′s chain shuttling polymerization technique. The chain shuttling polymerization employs two post-metallocene catalysts screened by high through-put technology and a chain shuttling agent, and the two catalysts have totally different incorporation abilities of α-olefin, thus producing different chain block, soft and hard PE segments, by a chain shuttling agent (diethyl zinc), and the produced chains are composed of at least two alternating soft and hard segments [8,26]. This type of multiblock chain structure gives the materials better elasticity at high temperature than their random counterparts [27].

In this work, a comparative analysis on the microstructures of these polyolefin-based elastomers was made by GPC, ^13^C NMR and TREF techniques. Then the toughening effects of these PBEs in the PP/PBE blends were investigated to evaluate the overall performance and toughening mechanism. A stiffness–balance–transparency relationship among the PP/PBE blends was established by the mechanical and thermal properties and the crystalline and rubber phase structure analysis.

## 2. Materials and Method

### 2.1. Materials

High-flow homopolypropylene (H9018H) provided by Lanzhou Petrochemical Company (PetroChina, Lanzhou, China) was employed in this work. Three types of polyolefin-based elastomers (PBE) for toughening were used to blend with homo-PP, including one POE sample, two OBC samples and one propylene-based elastomer sample. The POE (ENGAGE 8200) was purchased from a distributor of Dow Chemical (SCG Chemicals, Bangkok, Thailand) and two OBCs (INFUSE 9100 and 9500) were purchased from Dow Chemical (Midland, Michigan, USA) as pellets, and the PBE (Vistamaax 6202) was purchased from ExxonMobil Company (Singapore) as pellets. Engage 8200 is an ethylene/1-octene random copolymer produced by constrained geometry catalyst (CGC) technique from Dow Chemical and designated as PBE-1. Infuse 9100 and 9500 are ethylene/1-octene multiblock copolymers produced by Dow Chemical′s Chain Shuttling Polymerization technique [5,6], and are designated as PBE-2 and PBE-3, respectively. Vistamaxx 6202, designated as PBE-4, is an ethylene/propylene random copolymer produced by ExxonMobil′s metallocene catalyst technique.

### 2.2. Sample Preparation

The PP/POE, PP/OBC and PP/PBE (90/10 weight percent for all samples) blends were extruded at 190 °C using a twin screw extruder (ZSE-34, LEISTRTIZE, Wiesbaden, Germany). The roller speed was 80 rpm and the feed speed was set as 20%. Standard testing specimens for notched Izod impact, flexural and tensile tests were prepared by an UN-100 Injection machine (Liuzhou Injection Molding Machinery, Liuzhou, China) at an injection temperature of 200 °C, mold temperature of 40 °C and injection pressure of 25 MPa. The PP testing samples were also prepared in the same procedure for comparison.

### 2.3. Characterization

#### 2.3.1. Mechanical Test

The notched Izod impact test was conducted on 23 °C and on a 92T Pendulum impact tester (TINIUS OLSEN, Philadelphia, PA, USA) with a hamper energy of 2.0103 kJ/m^2^ according to GB/T 1843-2008. An Instron 5566 universal testing machine (Instron, Norwood, MA, USA) was used to test the tensile and flexural performances at room temperature (23 °C) according to standards of GB/T 1040.1-2006 and GB/T 9341-2008, respectively. The melting index was conducted on a CEAST 7028 (CEAST, Turin, Italy) according to GBT 3682, and the heat distortion temperature is tested on XRW-300 (Jingjianjiance, Chende, China) according to GB/T 1634.1-2004. Before the test, all test specimens were kept in 23 °C for 24 h, and the test result was an average value.

#### 2.3.2. GPC and ^13^C NMR Analysis

Gel permeation chromatography (GPC) was conducted in a GPC-IR instrument (Polymer Char, Valencia, Spain) using 1,2,4-trichlorobenzene as a solvent at 135 °C with a sampling concentration of 3 mg/mL and a sampling rate of 1.0 mL/min. ^13^C NMR spectra of samples were performed on a Bruker (Breika, Massachusetts, USA) 500 MHz at 120 °C using *o*-C_6_H_4_Cl_2_/*o*-C_6_D_4_Cl_2_ (50% *v*/*v*) as a solvent in a 10-mm tube. The spectra of the quantitative ^13^C NMR were obtained with a 74° flip angle, an acquisition time of 1.5 s, and a delay of 4.0 s.

#### 2.3.3. DSC and XRD Analysis

Differential scanning calorimetry (DSC) measurements were performed on a DSC 214 Polyma instrument (NETZSCH, Selb, Germany). In order to eliminate the heat history of the sample, a 7-mg sample was heated from room temperature to 200 °C under nitrogen protection at a heating rate of 30 °C /min, then cooled to 30 °C at a cooling rate of 20 °C /min. The melting curves were obtained when heating the samples to 200 °C at the same heating rate once again, and crystallization curves were recorded when cooling the samples to 30 °C. Wide-angle X-ray diffraction (WXRD) was performed on a D8 ADVANCE diffractometer (Bruker, Karlsruher, Germany) using a 1-mm-thick sheet of samples. The samples were scanned at 40 °C and 4°/min under Cu-Kα irradiation (λ = 0.154 nm). 

#### 2.3.4. TREF

Temperature rising elution fractionation (TREF) was carried out in a model 200+ instrument (Polymer Char, Spain). Standard Conditions were used in all analyses with 40 mg in 20 mL of 1,2,4-trichlorobenzene (TCB), 0.3 mL analysis sample volume, crystallization rate of 0.5 °C/min, and elution rate of 1 °C/min.

#### 2.3.5. Morphology Observation

Isothermal crystallization was observed using a polarized optical microscope, (DM2500P, Leica, Weztlar, Germany). The specimens for SEM observation were prepared by the cryogenic fracture of the injection molded bars under liquid nitrogen for 15 min and then etched in xylene at room temperature for 24 h for rubber phase removal. The fracture surface morphology was observed on a ULTRA plus field-emission electron microscope (FESEM, Zeiss, Oberkochen, Germany), after being coated with a thin layer of gold-palladium in vacuum. 

## 3. Results and Discussion

### 3.1. Polyolefin-Based Elastomers

**Figure 1:**
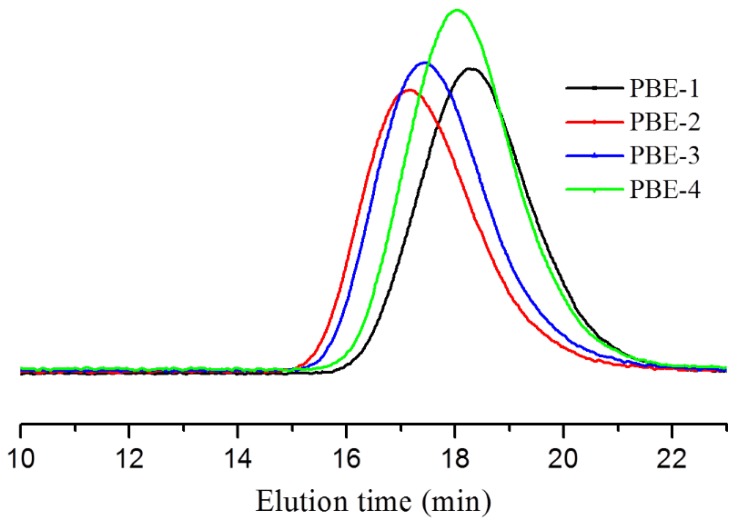
GPC molecular weight distribution comparison of Polyolefin-based elastomers.

The high temperature GPC was used to determine their molecular weights (*M*_w_ and *M*_n_) and polydispersity index (PDI). We can see from the GPC elution curves (Figure 1) that the ethylene/1-octene random copolymer (PBE-1) has the longest elution time which means it has the lowest average molecular weight compared to other elastomers. The average molecular weight of the propylene-based ethylene/propylene copolymer (PBE-4) is close to that of PBE-1. Due to the single-site nature of metallocene catalysts, PBE-1 and PBE-4 have a narrower molecular weight distribution (close to 2) compared to conventional Z-N catalysts. The olefin multiblock copolymers PBE-2 and PBE-3 have shorter elution times and, therefore, have a much higher molecular weight. Via the chain shuttling polymerization techniques in a continuous process, the molecular weight distributions of PBE-2 and PBE-3 can also obtain narrow distribution close to Schulz-Flory distribution (*M*_w_/*M*_n_ = 2) compared to other multi-sites Z-N catalysts, with 2.9 and 2.7 of PDI, respectively [8,28,29]. The molecular weight and PDI data are listed in Table 1.

**Figure 2:**
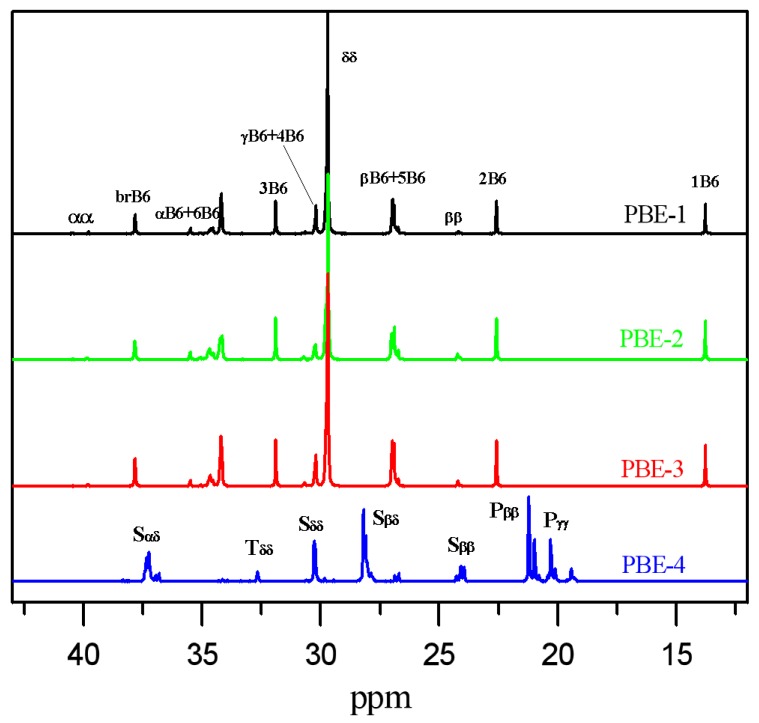
^13^C NMR spectrum of polyolefin-based elastomers measured at 120 °C using *o*-C_6_H_4_Cl_2_/*o*-C_6_D_4_Cl_2_ (50% *v*/*v*) as the solvent.

As shown in Table 1, comonomers content in the polyolefin-based elastomers do not vary much, roughly from 12% to 18%, with 12.2% 1-octene in the ethylene/1-octene random copolymer (PBE-1), 18.2% and 15.1% 1-octene in the olefin multiblock copolymers PBE-2 and PBE-3, respectively, and 16.5% ethylene in the propylene-based ethylene/propylene copolymer (PBE-4). The microstructure differences of the PBEs could be more clearly exhibited from the ^13^C NMR results in Figure 2 and Table 2. Table 2 provides the sequence fractions of the polyolefin-based elastomers obtained from the ^13^C NMR spectra [30,31,32,33]. From the NMR results, there is no OOO triad sequence detected in PBE-1, PBE-2 and PBE-3. Only a small amount of OO diad sequence about 0.8% exist in ethylene/1-octene copolymers PBE-1 and PBE-3, and PBE-2 has about 1.6% OO diad sequence. 

Along the NMR, TREF is a useful tool to compare the molecular chain microstructure of these PBEs. As seen from the TREF curves (Figure 8a), the PBE-1 and PBE-4 are typical ethylene/1-octene or ethylene/propylene random copolymers with a major soluble peak, while PBE-2 and PBE-3 are typical olefin block copolymer with characteristic multiblock peaks around 80–100 °C, which is different from linear low density polyethylene (LLDPE) or high density polyethylene (HDPE), apart from the soluble peaks. Comparing the two OBCs (PBE-2 and PBE-3), we found out that PBE-3 has higher soluble fraction than PBE-2 with less 1-octene content, and that could be explained by a higher content of multiblock peaks around 80–100 °C of PBE-2 observed in TREF curves.

### 3.2. PP/Polyolefin-Based Elastomer Blends

**Figure 3:**
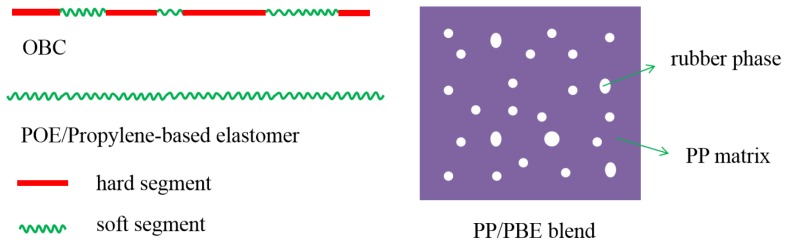
Scheme of molecular chain and aggregation structure of PP/polyolefin-based elastomer blends.

Three types of polyolefin-based elastomers including POE (PBE-1), OBC (PBE-2 and PBE-3) and propylene-based ethylene-propylene copolymer (PBE-4) were used to blend with polypropylene. The molecular structures of the three elastomers are shown in Figure 3. The molecular chain structure of OBC consists of hard and soft segments with different lengths, while the whole molecular chain of POE and propylene-based elastomer mainly consists of soft segments, which can be etched by xylene in room temperature and shown as the rubber phase in the PP/PBE blend.

**Figure 4:**
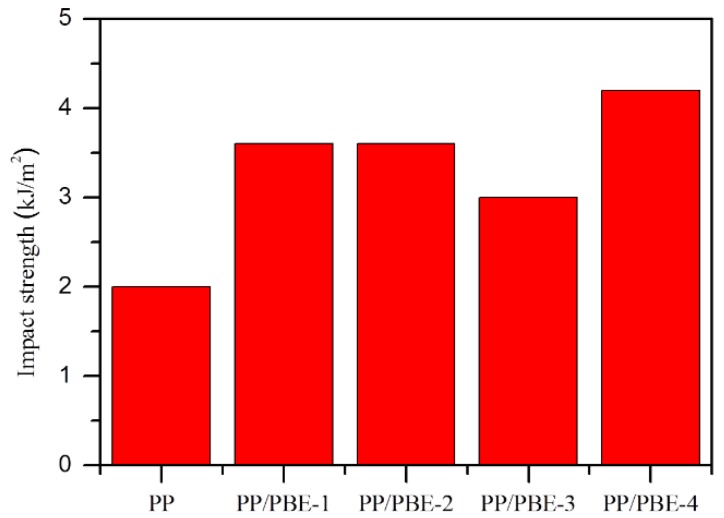
The notch Izod impact strength for PP and PP/PBE blends tested at 23 °C.

To evaluate the overall performance of these PBEs, the mechanical and optical properties of the PP and PP/PBE blends were tested, and the results are shown in Table 3. From Table 3, we can see the three types of PBEs take a different blending effect on the PP/PBE blends. On one hand, all three PBEs have a positive consequence on the toughening effect of the PP/PBE blends at the cost of the stiffness of the blends. The flexural modulus of the PP is the highest at 1850 MPa, while the impact strength is the lowest at 2.0 kJ/m^2^. Therefore, there is a need to toughen PP to improve the overall performance of the final PP product. As seen from Figure 4, all of the four PBEs have efficiently improved the toughness of PP blends. PBE-3 (OBC) raised the impact strength from 2.0 to 3.0 kJ/m^2^ with a 50% increase; PBE-1 (POE) and PBE-2 (OBC) increased the impact strength to 3.6 kJ/m^2^, an 80% increase; and PBE-4 increased the impact strength to 4.2 kJ/m^2^, a dramatic 110% increase. The different toughening effects could be due to the different molecular chain structures as seen in Figure 3.

As for the overall performance of the final PP product, the balance between stiffness and toughness needs to be taken into consideration. Despite the excellent toughening effect of PBE-4, the flexural modulus and the heat distortion temperature decreased rapidly at the same time. It would not be sufficient for use as a heat-resistant food container with 1540 MPa of flexural modulus and 96 °C of HDT. By the same token, the 3.0 kJ/m^2^ of impact strength for PBE-3 might not be good enough for impact resistance. The PBE-1 and PBE-2 have a good balance between stiffness and toughness with about 1700 MPa flexural modulus, about 110 °C HDT and 3.6 kJ/m^2^ impact strength, which would be suitable for the use mentioned above.

The tensile yield stress of the PBEs has a similar trend to the flexural modulus on the PP/PBE blends as seen in Table 3. The increases of the elongation at break are mainly due to the addition of the polyolefin-based elastomers containing soft segment molecular chains.

On the other hand, the optical properties sometimes also need to be taken into account for the appearance aspect. The haze results are list in Table 3. Although PBE-1 and PBE-2 have a great stiffness–toughness balance on the final PP/PBE blends, the optical properties, especially the haze, increased notoriously from 71% to 83% and 98% respectively, causing bad transparency. Furthermore, the PBE-3 also has a similar influence on the PP/PBE-3 blend as it has the same type of elastomer as PBE-2 (OBC), resulting in an opaque appearance with 99% of haze. In contrast, PBE-4 has the best effect on the transparency of the PP/PBE blends, decreasing the haze from 71% to 57%, despite leading to bad stiffness.

**Figure 5:**
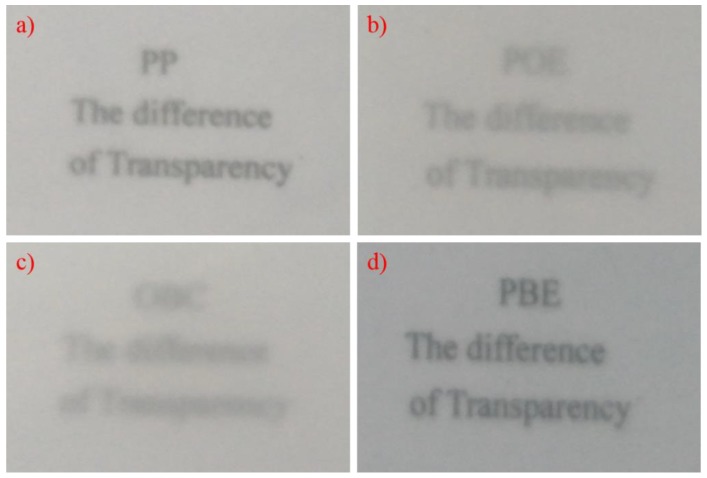
Digital photos of transparency comparison of PP and PP/PBE blends of 2-mm sheet. (**a**) PP; (**b**) PBE-1; (**c**) PBE-2; (**d**) PBE-4.

The transparency comparison can be clearly illustrated from the digital photos of the PP/PBE blends in Figure 5. The text can be read from the 2-mm PP sheet, it is illegible from the PP/PBE-1 sheet, and it is hard to identify for the PP/PBE-2 sheet, while the PP/PBE-4 provided a higher resolution for the text. For this reason, the PP/PBE-4 blends would be an excellent material for transparent and impact-resistant use, if not used in relatively high temperatures.

### 3.3. XRD Analysis

**Figure 6:**
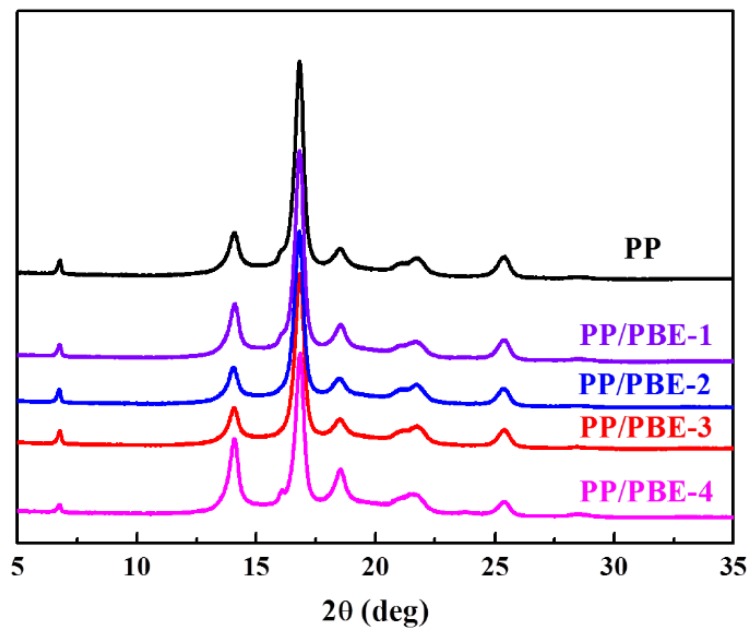
XRD of PP and PP/PBE blends.

Wide angle X-ray diffraction analysis were conducted to analyze the PP crystal type and content. From Figure 6, the typical diffuse peaks of α crystal of PP can be observed with the 2θ of 14.1°, 16.8° and 18.6°, which are attributed to the (110), (040) and (130) crystal faces, respectively, and these peaks also appears in the other four PP/PBE blends indicating that the major PP crystal type is α crystal. The tiny characteristic peaks of β crystal could be seen in PP and the four PP/PBE blends with slight peaks either around 16.1° or 21.1°, which contribute to the (300) and (301) crystal faces, respectively, indicating that PP and the PP/PBE blends contain slight β crystals. The peak around 20.5° is unobserved, which shows that the γ crystal in PP and PP/PBE blends could be neglected. 

The relative crystallinity index from WAXD of PP and the PP/PBE blends were calculated in Table 3. The crystallinity of PP is 63.6%, followed by PP/PBE-3 of 62.1%, PP/PBE-1 of 60.9%, PP/PEB-2 of 59.3%, and PP/PBE-4 of 58.8%. The stiffness (Flexural modulus and HDT) of PP and PP/PBE blends are roughly in accordance with the crystallinity of the polymers. It would make sense that crystal content is linear to stiffness with α formation as a major crystal. The polyoelfin-based elasomers used in the PP/PBE blends make the PP spherulite size smaller as seen in the polarized optical microscope, and thus decrease the crystallinity of PP; we will discuss the factor of crystal size in the morphology section later.

### 3.4. DSC Analysis

**Figure 7:**
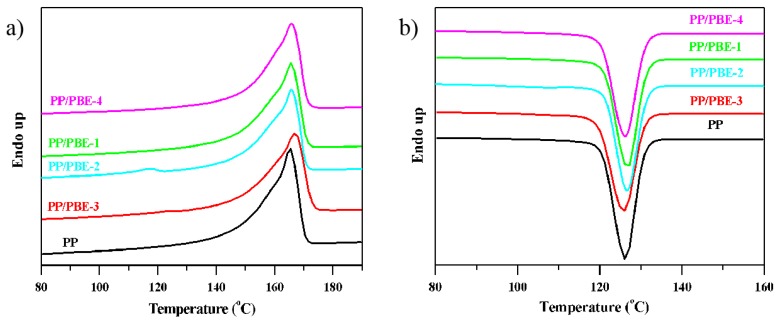
DSC curves of PP and PP/PBE blends. (**a**) melting curves; (**b**) cooling curves.

As is well known, the thermal properties are strongly related with the crystallinity of the materials and their microstructure. The DSC thermal analysis was carried out to exhibit the melting and crystalline behavior. As seen from Figure 7a., the melting curve of PP/PBE-2 has two peaks. The peak around 166 °C is attributed to the melting peak of the isotactic homo-PP, and the other distinctive peak is due to the crystalline part of OBC containing both hard and soft segments in the molecular chain, with the melting peak around 117 °C for the PP/PBE-2 blend. However, only one peak was visible in the PP/PBE-3; the absence of the minor peak around 120 °C from the characteristic melting peak of OBC might be due to the relatively low content of the soft-hard segment alternating OBC chain (see the evidence from TREF results). From Figure 7b, a slightly shift to the right or a higher temperature range of the starting point of crystallization temperature *T*_c_ (onset) can be noticed with the PP/PBE blends compared to PP, signifying that the nucleating speed of the PP spherulite increased and crystal size became smaller when the polyolefin-based elastomers were used.

The melting temperature *T*_m_, crystallization temperature *T*_c_ and the melting enthalpy of PP and the PP/PBE blends from DSC analysis results are shown in Table 4. The crystallization peak of PP and the PP/PBE blends are around 126 °C, and the melting endothermic enthalpy of the PP/PBE blends diminishes in contrast to PP. The melting endothermic enthalpy Δ*H*_m_ can be used to calculate the relative crystallinity *X*_c_, thus the value of Δ*H*_m_ is a direct indication of the degree of crystallinity with the highest *X*_c_ for the PP/PBE-3 and the lowest *X*_c_ for PP/PBE-4. This melting enthalpy comparison of these materials is similar to the trend in the XRD results.

### 3.5. TREF Analysis

**Figure 8:**
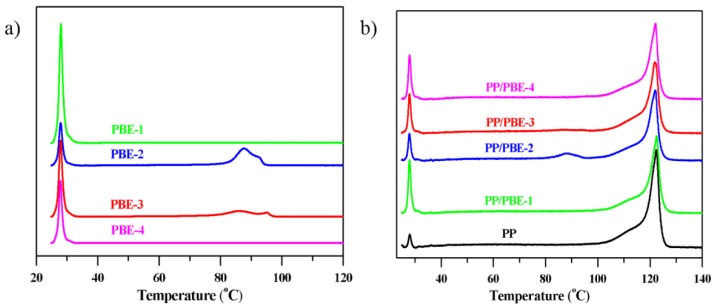
TREF curves. (**a**) Four polyolefin-based elastomers (PBEs); (**b**) PP and PP/PBE blends.

To explore the microstructure of the polyolefin-based elastomers and the toughened PP/PBE blends, the TREF technique was adopted to characterize the Chemical Composition Distribution (CCDs) of these materials. As reasoned before, Figure 8a shows the TREF analysis of three types of polyolefin-based elastomers (PBE-1/PBE-2 and PBE-3/PBE-4). In the POE (PBE-1) curve, only a soluble fraction peak appears, which is attributed to the ethylene/1-octene (E/O) random copolymer. While in addition to the SF peak, extra peaks in the higher temperature range (80–100 °C) exist in the curves of OBCs (PBE-2 and PBE-3). The CCDs of SF in OBC are supposed to be similar with the SF in the POE and basically consist of an ethylene/1-octene random copolymer (soft segment); the CCDs in the higher temperature are due to the molecular chain with alternating hard and soft segments produced by the chain shuttling polymerization technology. The hard segment is composed of a polyethylene chain with a trace 1-octene comonomer, if any scattered in the chain, and the soft segment as mentioned above is composed of an ethylene/1-octene random copolymer. Obviously, the chemical composition and CCDs vary in the two OBCs, and PBE-2 contains harder–softer segment alternating molecular chains. From the impact test results, we can reasonably infer that the hard–soft segment alternating molecular chains could possess as excellent a toughening effect as the ethylene/1-octene random molecular chain [34].

The third type of PBE is a propylene-based propylene/ethylene copolymer, and most propylene/ethylene (P/E) copolymer chains are soluble, which indicates that the PBE-4 consists basically of the E/P random copolymer. As seen in Table 5 of the TREF analysis results, PBE-4 contains 93.5% soluble fraction, and only a small part of the fractions in 58, 64 and 73 °C might be ascribed to higher molecular chain regularity of PPPP (mmm) sequence distribution, which could be discovered in a 46.5 ppm small peak in the ^13^C NMR of PBE-4 [32,33]. At the same time, the PPPPP (mmmm) sequence in 21.8 ppm in the ^13^C NMR is not observed, which is in compliance with the absence of a peak around 120 °C of isotactic PP of TREF analysis.

From Figure 8b, we can identify the highly isotactic homo-polypropylene around 122 °C in PP and the PP/PBE blends. Except for the highly isotactic PP of 87% in the homo-PP, there is a small peak of SF of 3.1% (see Table 5) for atactic PP, and other CCDs with less regularities of PP chain structures around 52, 66 and 89 °C are also listed in Table 5, despite not obviously being in these zones. The PP/PBE-1 blend has 12.6% of SF, and this value is high above the SF from PP, which is attributed to the atactic PP and E/O random copolymer from POE. Similarly, part of the SF of the PP/PBE-2 and PP/PBE-3 blends belong to the soluble E/O random copolymer. Moreover, the peak at 88 °C in the PP/PBE-2 blends and peaks at 87 and 93 °C in the PP/PBE-3 blends are mainly due to the alternating hard and soft segments from PBE-2 and PBE-3, respectively. As for the PP/PBE-4 blends, the E/P random copolymer compositions account for the increased soluble fraction.

### 3.6. Morphology Analysis

**Figure 9:**
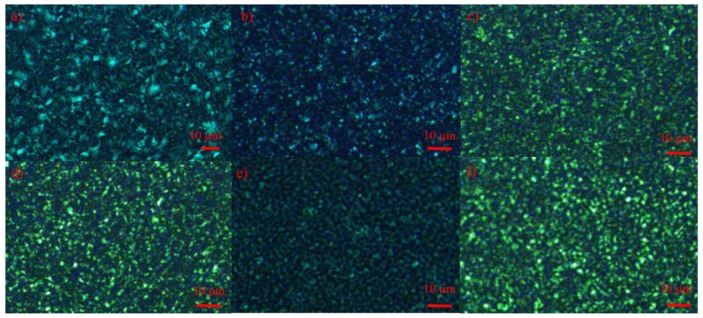
Polarized optical microscope photos of PP and PP/PBE blends in 150 °C stabilizing for 5 min, (**a**) PP; (**b**) PP/PBE-1; (**c**,**d**) PP/PBE-2; (**e**) PP/PBE-3; (**f**) PP/PBE-4.

**Figure 10:**
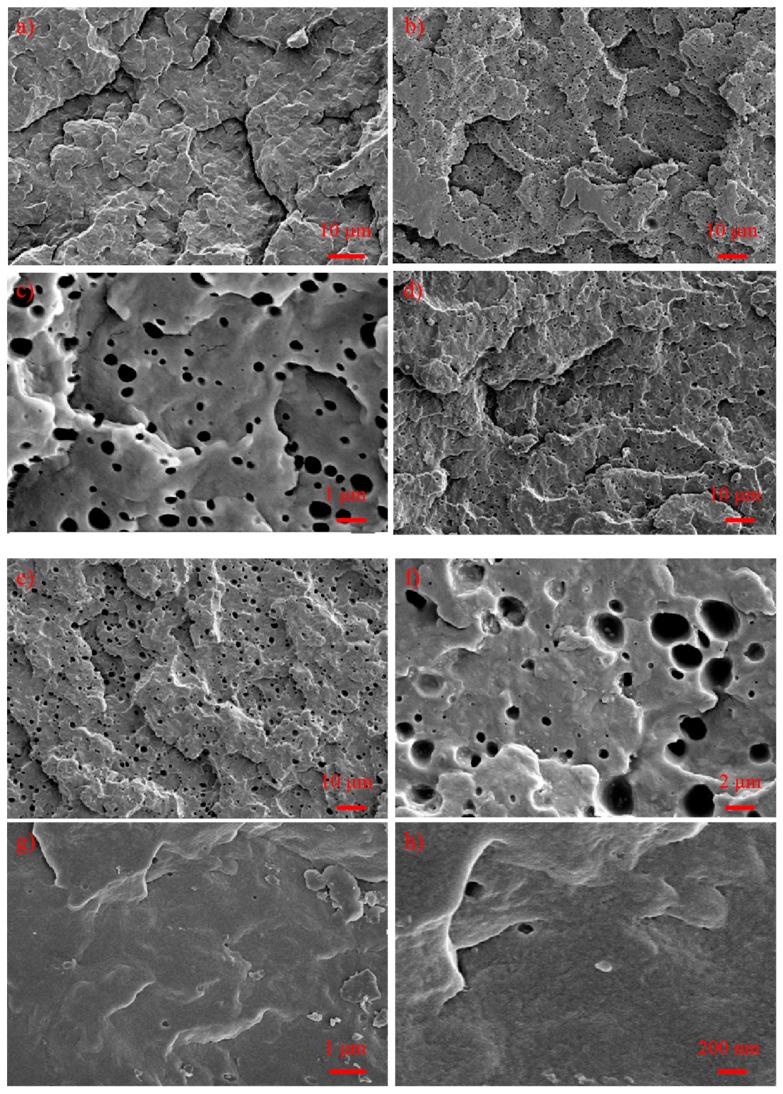
Scanning electron microscope imaging of PP and PP/PBE blends; (**a**) PP; (**b**,**c**) PP/PBE-1; (**d**) PP/PBE-2; (**e**,**f**) PP/PBE-3; (**g**,**h**) PP/PBE-4.

In order to evaluate the toughening and optical effects of these polyolefin-based elastomers on the PP/PBE blends, polarized optical microscope (POM) and scanning electron microscope (SEM) analysis would be quite effective for the investigation of the crystal and rubber size. As shown in Figure 9 and Figure 10, the spherulite size of PP is the biggest among these materials, and the spherulite size could be up to 10 μm; and there are seldom rubber phases in the PP matrix (the formed holes by xylene etching as seen from the SEM images), which leads to its poor impact performance. When these polyolefin-based elastomers were used to modify PP, the crystal size of their PP/PBE blends became smaller, and the rubber phase can be observed evidently in the PP/PBE-1, PP/PBE-2 and PP/PBE-3 blends. That is the main reason for toughening the PP matrix.

It would be beneficial to have a smaller crystal size for light transmission of these materials, however, the large size of the rubber in the PP/PBE-1, PP/PBE-2 and PP/PBE-3 blends, meanwhile, hinders the transmission of light. Generally, when the rubber size is above the wavelength of the visible spectroscopy (roughly 400–800 nm), the transmittance of the materials will drop and the haze will rise. In the PP/PBE-3 blend, the rubber size is up to about 2 μm, and the haze of the blend climbs to 99%.

Surprisingly, the rubber phase by xylene etching can be rarely observed in the PP/PBE-4 blend with less than 200 nm rubber apertures scarcely scattered in the cross section. This explains why the PP/PBE blend has better transparency for visible spectroscopy with a smaller crystal size. Furthermore, as can be inferred, the propylene-based E/P copolymer has good compatibility with the isotactic homo-PP molecular chain, forming no apparent phase separation; therefore, this great compatibility also results in a distinctive toughening effect.

## 4. Conclusions

The molecular chain structures of the polyolefin-based elastomers were thoroughly studied by GPC, ^13^C NMR, TREF, and DSC techniques. Despite the different microstructures, all three types of PBEs can effectively improve the toughness of the PP/PBE blends by the addition of the rubber compositions. PBE-1 and PBE-2 have great stiffness–toughness balance with about 1700 MPa of flexural modulus, about 110 °C of HDT and 3.6 kJ/m^2^ of impact strength on the prepared PP/PBE blends by forming separated rubber phase and refined spherulite crystals. The toughening mechanism of the PBEs were further investigated by TREF, DSC, XRD, POM, and SEM. The results showed that the rubber size has a significant influence on stiffness and optical properties of the PP/PBE blends. The PBE-2 with alternating hard and soft segments could achieve a similar toughening effect as the E/P random copolymer (PBE-1) when a similar sized rubber phase was formed. Unexpectedly, no obvious rubber phase was observed in the PP/PBE-4 blend. Due to the excellent compatibility of the E/P random chains with the isotactic PP, the PP/PBE blend obtained great toughness performance and optical transparency with the highest Izod impact strength of 4.2 kJ/m^2^ and excellent transparency. This is significantly important for the research and development of high-performance novel PP products with great stiffness–toughness balance or transparent and impact-resistant PP for industrial applications.

## Figures and Tables

**Table 1:** Characteristics of the used polyolefin-based elastomers.

Entry	Sample	Mol% ^a^ Comon.	*M*_w_^b^ (kg/mol)	*M*_n_ (kg/mol)	PDI
1	PBE-1	12.2 (1-octene)	74.2	31.7	2.3
2	PBE-2	18.2 (1-octene)	178.2	62.1	2.9
3	PBE-3	15.1 (1-octene)	138	51.6	2.7
4	PBE-4	16.5 (ethylene)	89.2	38.7	2.3

^a^ Molar fraction of comonomers in elastomers determined by NMR; ^b^ Determined by GPC.

**Table 2:** The triad and diad distributions of ethylene/1-octene and ethylene/propylene copolymers obtained by ^13^C NMR.

Sample	EEE (%)	EEO + OEE/EEP + PEE (%)	OEO/PEP (%)	EOE/EPE (%)	EOO + OOE/EPP + PPE (%)	OOO/PPP (%)	EE (%)	EO + OE/EP + PE (%)	OO/PP (%)
PBE-1	66.05	20.66	1.62	10.08	1.59	0	76.38	22.82	0.80
PBE-2	54.27	21.76	6.03	14.67	3.27	0	65.15	33.12	1.63
PBE-3	60.26	20.54	3.41	14.25	1.54	0	70.53	28.7	0.77
PBE-4	0.03	4.25	11.31	2.76	25.13	56.52	2.15	28.77	69.08

**Table 3:** Mechanical analysis results of the PP and PP/PBE blends.

Entry	^f^ Sample	Melting Index g/10 min	Flexural Modulus MPa	Tensile Yield Stress MPa	Elongation at Break %	Impact Strength kJ/m^2^	HDT °C	Haze (1 mm Sheet) %	*X*_c_, WAXD %
5	PP	73	1850	37.8	8.1	2.0	119	71	63.6
6	PP/PBE-1	53	1690	35.5	14.1	3.6	112	83	60.9
7	PP/PBE-2	53	1680	35.5	10.2	3.6	109	98	59.3
8	PP/PBE-3	55	1750	36.8	9.3	3.0	113	99	62.1
9	PP/PBE-4	54	1350	33.0	12.7	4.2	96	57	58.8

^f^ PP/PBE (1/2/3/4) samples were mixed and melting extruded on PP to PBE (1/2/3/4) weight ratio 90:10, respectively.

**Table 4:** DSC analysis results.

Sample	*T*_c_ (Peak) °C	*T*_c_ (Onset) °C	*T*_m_ (Peak) °C	Δ*H*_m_ J/g
PP	126.2	130.7	165.3	151.4
PP/PBE-1	126.8	131.3	165.7	134.4
PP/PBE-2	126.7	131.4	165.8	129.3
PP/PBE-3	126.1	130.8	167.1	139.9
PP/PBE-4	126.2	131.1	165.9	128.9

**Table 5:** TREF analysis results.

Sample	Item	Soluble Fraction (SF)	Peak 1	Peak 2	Peak 3	Peak 4
PBE-1	T/°C		-	-	-	-
	Area/%	100	-	-	-	-
PBE-2	T/°C		-	-	87.7	-
	Area/%	34.1	-	-	65.9	-
PBE-3	T/°C		-	86.0	94.6	-
	Area/%	60.1	-	33.2	6.7	-
PBE-4	T/°C		58.0	64.0	73.3	94.1
	Area/%	93.5	3.9	1.1	0.8	0.8
PP	T/°C		52.2	65.9	89.3	122.0
	Area/%	3.1	4.3	3.1	2.5	87.0
PP/PBE-1	T/°C		51.2	66.3	93.2	122.1
	Area/%	12.6	1.7	1.4	2.5	81.8
PP/PBE-2	T/°C		50.1	73.5	88.1	121.5
	Area/%	6.3	1.5	1.9	13.6	76.6
PP/PBE-3	T/°C		60.8	86.9	93.5	121.7
	Area/%	9.8	1.9	6.9	2.5	78.9
PP/PBE-4	T/°C		53.1	67.6	86.8	121.6
	Area/%	10.6	3.0	1.5	1.3	83.5
